# eDNA metabarcoding reveals the role of habitat specialization and spatial and environmental variability in shaping diversity patterns of fish metacommunities

**DOI:** 10.1371/journal.pone.0296310

**Published:** 2024-01-02

**Authors:** Tibor Erős, Andrea Funk, Didier Pont, Thomas Hein, Paul Meulenbroek, Bálint Preiszner, Alice Valentini, István Czeglédi

**Affiliations:** 1 HUN-REN Balaton Limnological Research Institute, Tihany, Hungary; 2 National Laboratory for Water Science and Water Security, HUN-REN Balaton Limnological Research Institute, Tihany, Hungary; 3 Christian Doppler Laboratory for Meta Ecosystem Dynamics in Riverine Landscapes, University of Natural Resources and Life Sciences, Vienna, Austria; 4 Institute of Hydrobiology and Aquatic Ecosystem Management, University of Natural Resources and Life Sciences, Vienna, Austria; 5 SPYGEN, Savoie Technolac, Le Bourget du Lac, France; University of Udine: Universita degli Studi di Udine, ITALY

## Abstract

Information is scarce on how environmental and dispersal processes interact with biological features of the organisms, such as their habitat affinity, to influence patterns in biodiversity. We examined the role of habitat specialist vs. generalist species, and the spatial configuration, connectivity, and different environmental characteristics of river-floodplain habitats to get a more mechanistic understanding of alpha and beta diversity of fish metacommunities. We used environmental DNA metabarcoding to characterize species (taxa) richness and composition in two separate floodplains of the river Danube (Austria and Hungary) during two different hydrological conditions. Results showed that differences in the number of generalist and specialist species and their responses to connectivity and environmental gradients influenced patterns in alpha and beta diversity. Of the components of beta diversity, richness difference (nestedness) showed consistently higher values than replacement (turnover), mainly due to the decrease of specialist species along the connectivity gradient (i.e., from the mainstem to the most isolated oxbows). Variance in both alpha and beta diversity could be well predicted by a set of local and regional variables, despite high environmental variability, which characterizes river-floodplain ecosystems. Of these, the joint or shared variance fractions proved to be the most important, which indicates that the effects of local and regional processes cannot be unambiguously separated in these river-floodplain systems. Local scale environmental variables were more important determinants of both alpha and beta diversity in the low water period than in the high water period. These results indicate the differential role of local and regional processes in community organization during different hydrological conditions. Maintenance of both local and regional scale processes are thus important in the preservation of alpha and beta diversity of floodplain fish metacommunities, which should be considered by environmental management.

## Introduction

A major goal of community ecology is to understand which factors control patterns in the diversity, composition and abundance of species [1]. Recent frameworks emphasize the role of both niche-based (or local) and dispersal related (or regional) processes in shaping the diversity of ecological communities [2–4]. In this approach the diversity of communities is examined in a metacommunity context, where sets of local communities are connected by the dispersal of several potentially interacting species. Such a regional scale view largely increased our ability to understand patterns in local (or alpha) and between site (or beta) diversity [5]. Overall, both empirical and model-based studies suggest that alpha and beta diversity can be predicted to some degree by considering the environmental characteristics of habitat patches and their connectivity relationships in the landscape [6, 7]. However, the role of local and regional processes may also depend on the response of organisms to environmental gradients, such as their habitat affinity and dispersal ability. For example, communities dominated by habitat specialist species may show a stronger response to local scale habitat features than communities where habitat generalists dominate [8]. Therefore, the ratio of habitat specialists and generalists can largely influence patterns in both alpha and beta diversity, and how patterns in diversity depend on local and regional processes.

Large floodplain rivers provide an ideal ecosystem type to study the relative role of local and regional scale processes on community organization. Floodplains are formed by a variety of freshwater habitat patches, which are surrounded by the terrestrial landscape. Environmental characteristics and connectivity relationships of these patches can vary largely depending on the dynamism of the water regime of the mainstem river. Floods can increase size and connectivity of the patches, which may increase habitat availability and diminish dispersal limitation for aquatic species. On the contrary, the environment may filter those species which are best suited to particular local habitat features in low flow periods. Consequently, the relative role of local and regional processes can vary largely depending on hydrological connectivity [9, 10]. Understanding the role of various processes that shape patterns in diversity is especially challenging in these dynamic systems, because they are hard to sample representatively, and there is considerable variability among regions and organism groups [11, 12]. Not surprisingly, it is less known how alpha and beta diversity change along different environmental and connectivity gradients, especially from relatively natural floodplains. Such knowledge could largely increase our ability to manage and protect the diversity of floodplain communities.

**Fig 1:**
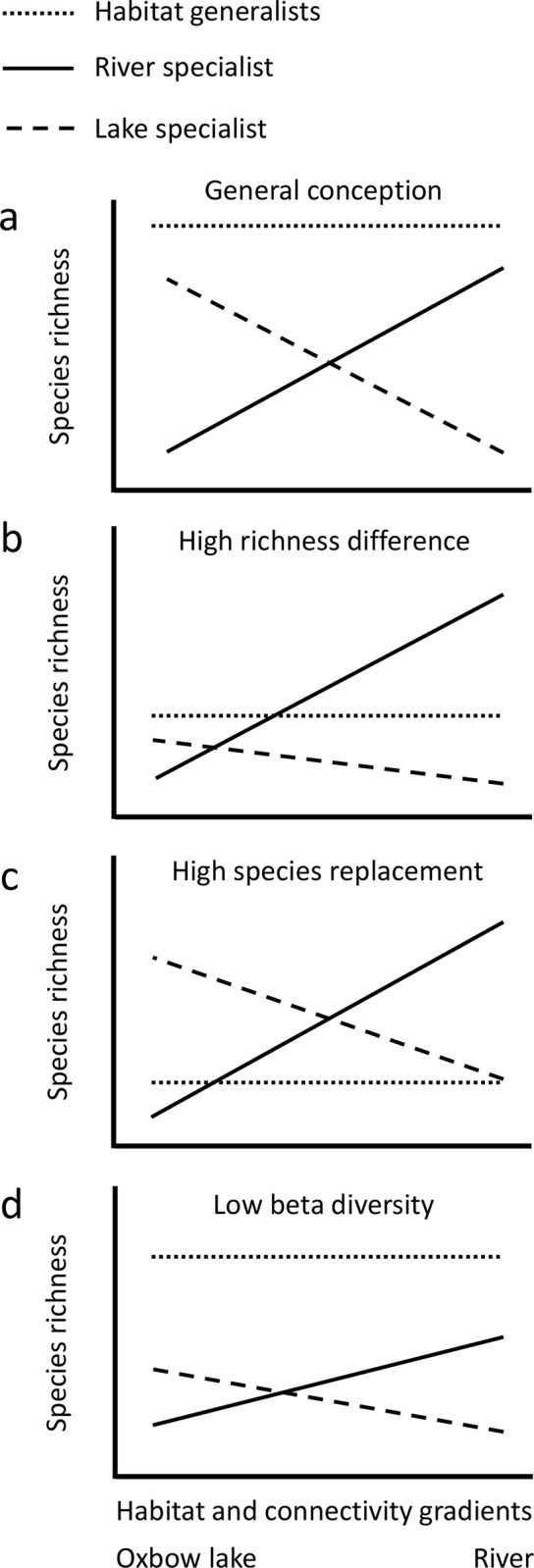
Predicted patterns in species richness for river-floodplain fish metacommunities. Examples show that alpha and beta diversity can depend largely on the length of environmental and connectivity gradients and the number and ratio of habitat specialist and generalist species (a). High richness difference or nestedness (sensu [15, 16] respectively) can occur, for example, in case of gradual loss of species from the mainstem river to the most isolated oxbow lakes, especially if the ratio of river specialists is high relative to habitat generalist and lake specialist species (b). On the contrary, high species replacement or turnover is expected if the number of both river and lake specialist species is high relative to the number of habitat generalist species (c). Beta diversity is low if the number of habitat generalist species is much higher than specialist ones (d). River and oxbow lake indicate the two endpoints of the habitat and connectivity gradients.

Given the lack of detailed knowledge on alpha and beta diversity of floodplain metacommunities and their determinants, here we examined patterns in the diversity of river-floodplain fish communities and the effects of local and regional scale processes on these patterns. A variety of responses in diversity has been shown for fish communities to connectivity and habitat gradients [13, 14]. Although how the ratio of habitat generalist and specialist species influence patterns in diversity is still largely unknown, it can be predicted that habitat affinity may substantially influence the responses of both alpha and beta diversity, and the components of beta diversity to environmental gradients (i.e., species replacement or turnover and richness difference or nestedness components, see e.g., [15–17] for definition; see Fig 1 for examples).

Here, we examine the diversity of fish communities in the floodplains of the river Danube during two different hydrological periods. We predicted that alpha diversity would decrease from the mainstem river, as source of the species pool, to the most isolated and smallest water bodies with zero connectivity. Therefore, we predicted gradual species loss from lotic to lentic habitats, which may indicate a nested pattern, and therefore, pinpoint to the importance of richness difference component in shaping beta diversity. Given the combined effects of habitat and connectivity gradients, we also predicted that both local and regional scale processes will jointly shape floodplain fish metacommunities. Specifically, we predicted that the joint or shared effect of environmental, connectivity, and spatial gradients will be a more significant determinant of fish assemblages than the independent or pure effects of these factor groups. Finally, we also predicted that the importance of regional scale variables (spatial and connectivity variables) will increase in the high water period compared to the low water period, when local scale environmental variables (i.e., land use, physical and chemical characteristics of the waterbodies) will be more important determinants of compositional changes.

Sampling fish communities with traditional gears (e.g., gillnets, fyke nets, electrofishing), may provide biased estimation of alpha and beta diversity, especially in large water bodies, where fully representative sampling is hard to conduct [18, 19]. Therefore, we use environmental DNA (eDNA), a non-invasive sampling method, which has been repeatedly shown to provide more representative samples than traditional ones in characterising species richness and composition of fish communities, especially in large rivers and their associated wetland habitats [20–23]. To our knowledge, this is the first study that quantifies alpha and beta diversity of floodplain fish metacommunities and their determinants along whole river-floodplain connectivity gradients using eDNA.

## Materials and methods

### Ethics statement

No animal was harmed, killed or suffered during the study, since we collected only water samples for eDNA. Field research was approved under permit no. PE-KTFO/5787-10/2020 issued by the National Department of Environment and Nature Protection.

### Study area

**Fig 2:**
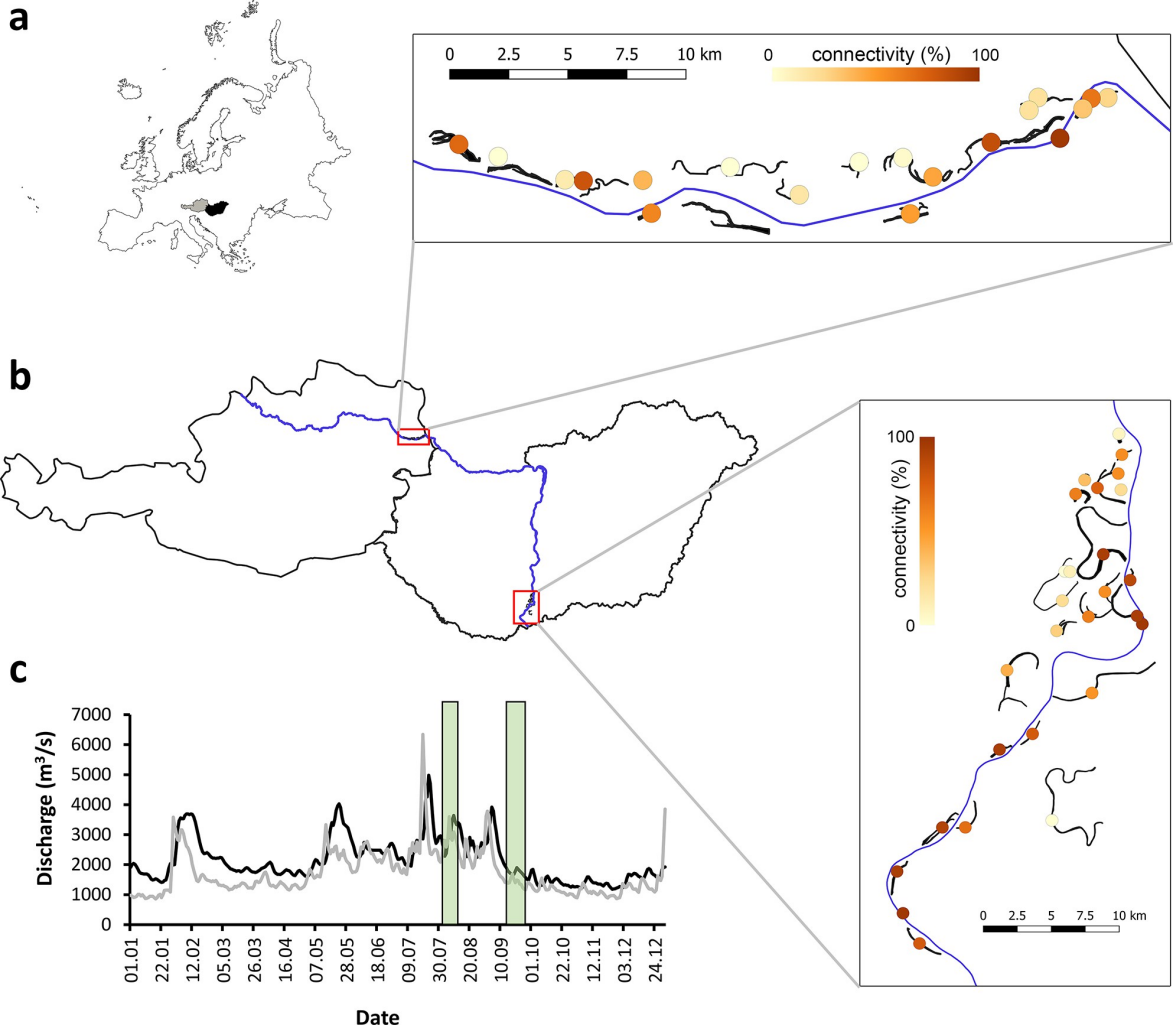
Map of the study area in Austria (grey) and Hungary (black) in Europe (a) with the studied water bodies and sampling locations (dots are coloured based on their connectivity value) (b). Characteristics of flow in Austria (grey line) and Hungary (black line) (c) in 2021 (the sampling period is indicated with green bar).

We studied two floodplains in the river Danube, Europe (Fig 2A and 2B). The Danube has a drainage area of approximately 800,000 km^2^ and a mean discharge of 6,500 m^3^ s^-1^ at its mouth. From source to mouth the Danube drains 19 countries, which makes the Danube basin the most international catchment in the world (http://www.icpdr.org/main/danube-basin). In the studied Danubian segment the mean annual discharge of the river is about 1900–2400 m^3^s^-1^. One floodplain is located in the Donau-Auen National Park in Austria (AUT), while the other one in the Danube-Dráva National Park in Hungary (HUN). Both floodplains contain the full variety of river-floodplain functional habitat types from side arms which are connected to the main channel at both ends to fully isolated oxbow lakes. The fish fauna (i.e., species pool) of the two studied systems is identical [24]. We examined the two floodplain systems during a high and a low water period, in the summer and early autumn of 2021, respectively (Fig 2C).

### Habitat specialization

We used the habitat affinity values of the floodplain fish index to distinguish habitat specialist vs. generalist species [25]. This index uses a fuzzy coding system (sensu [26]) to characterize the probability of occurrence of 163 European lamprey and fish species along the lateral connectivity gradient of floodplain rivers. Specifically, using species distribution data all over Europe the affinity of the species to five major river-floodplain waterbody types was scored between 0 and 10 for each type, summing up to a total of 10 across all types [25]. In general, specialist species occur only in a restricted amount of habitat types and can be characterized with high affinity scores to these habitats, while generalist species occur along the full gradient of floodplain habitats with low affinity scores. For this study, we defined specialist species those that occur in a maximum of three floodplain water body types, but their affinity value in the third habitat type cannot be higher than 2 (20%). This practically means that specialist species occur in just one end of the habitat connectivity gradient (i.e., occur mainly in one or two habitat types with high affinity scores). All species which did not fulfil this requirement were considered as generalist. For the categorization of species/taxa see S1 Table. Note that we found only one lake specialist species (mudminnow, *Umbra krameri*) during our surveys, and therefore we conducted the analyses only with riverine specialists, beside habitat generalists.

### Environmental and spatial data

Waterbodies in the floodplains were delineated using aerial photographs and a digital elevation model (DEM) based on inflow areas and points of intersections within the network (see also [27, 28]). Altogether 19 and 27 sites were studied in AUT and HUN, respectively (Fig 2B). The sites represented all types of functional habitats from the main river to most isolated oxbows.

Information on land-use within a 500 m wide buffer area around the water bodies was obtained from the CORINE Land Cover 2018 database (European Environmental Agency, 2020, http://www.eea.europa.eu). The following five robust categories were used for the characterization of land use: % agricultural, % forest and other seminatural habitats, % pasture, % urban areas, % water and wetland. Hydrological connectivity was defined as % mean number of days in a year a waterbody is connected to the main channel [27, 28]. Areas of the waterbodies were measured using GIS tools. Mean depth and mean current velocity were measured using a digital terrain model and a water velocity meter, respectively. Specifically, for mean depth elevation data were based on a digital terrain model (DTM) derived from a laser scan supplemented by bed elevation measurements from deeper side arms. Surface water levels in Austria were derived from the Austrian River Authority (via Donau Österreichische Wasserstraßen GmbH) and in Hungary from the National Water Authority. Within waterbodies, habitat structure was further characterized using visually estimated percentage composition of the following variables at the place of the samplings: % emergent, % submerged and % floating vegetation, % floating algae, % open water habitat, % woody debris. The bank structure was similarly characterized using the following variables: % woody (i.e., tree or large bushes), % herbaceous, % artificial (concrete, rip-rap). % canopy cover was also estimated. Substratum types were grouped into silt, sand, gravel and rock classes based on the particle size, and their percentage cover was estimated. Physicochemical variables were either measured in the field using portable sensors (pH, temperature°C, conductivity μS cm^-1^, dissolved oxygen mg L^-1^; Hach-Lange HQ40) or in the laboratory using water samples taken in the field (dry weight mg L^-1^, ash weight mg L^-1^, total dissolved phosphorus μg L^-1^, chlorophyll-a μg L^-1^, CDOM—humic Pt colour mg L^-1^). To quantify chlorophyll-a 300–2000 mL of sample water was filtered through pre-combusted GF/C filters (Whatman). Chlorophyll-a was extracted in cold acetone (90%) and the absorption was measured using a spectrophotometer [29, 30]. Total P was digested with persulfate according to [31] and analysed complying with ÖNORM EN ISO 15681–2. To quantify total suspended solids (TSS) and particulate organic matter (POM) 300–2000 mL of sample water was filtered through pre-combusted GF/F filters (Whatman). For TSS the filters were dried at 80°C for 24h. TSS was determined by weighing. POM was determined after ashing (450°C, 4h). CDOM was measured following [32] by filtering water with 0.45 μm Teflon membrane filters and measuring light absorbance at 440 nm. For the general characteristics of the sites and the environmental variables used for the data analyses, see S2 Table.

### eDNA metabarcoding analysis

Water samples for eDNA metabarcoding analyses were collected either from a boat or by wading slowly crossing the available mesohabitats at each floodplain site. To avoid eDNA cross-contamination among sites, when entering in the water, the operator remained downstream from the filtration area by holding the sampling device and showing directly towards the movement of the boat or direction of walking. Water was filtered in situ via a VigiDNA 0.45 μm crossflow filtration capsule (SPYGEN) using a peristaltic pump and a disposable sterile tubing attached to a rod covered with a single-use sterile plastic protection and pointed in the direction of progression. One filter was used per site and session and the mean water volume filtered was 18.31 L (3 to 94 L), depending on the clogging speed of the filtration capsule. Note that preliminary investigations showed no relationships between the volume of water sampled and the number of species/taxa in this system (Pearson correlation, R^2^ = 0.006, P = 0.978) [26]. At the end of each filtration, the water in the capsule was drained and the capsule was refilled with 80 mL of conservation buffer CL1 (SPYGEN) to prevent eDNA degradation. For the DNA extraction, each filtration capsule was agitated on an S50 shaker (Ingenieurbüro CAT M.Zipperer GmbH, Ballrechten-Dottingen, Germany) at 800 rpm for 15 min, dec-anted into a 50 mL tube, and centrifuged at 15,000 × g and 6°C for 15 min. The supernatant was removed with a sterile pipette, leaving 15 mL of liquid at the bottom of the tube. Subsequently, 33 mL of ethanol and 1.5 mL of 3 M sodium acetate were added to each 50 mL tube, and the mixtures were stored at −20°C for at least one night. The tubes were then centrifuged at 15,000 × g and 6°C for 15 min, and the supernatants were discarded. Then, 720 μL of ATL buffer from a DNeasy Blood & Tissue Extraction Kit (Qiagen, Hilden, Germany) was added. The tubes were vortexed, and the supernatants were transferred to 2 mL tubes containing 20 μL proteinase K. The tubes were then incubated at 56°C for 2 h. DNA extraction was performed using a NucleoSpin Soil kit (Macherey-Nagel GmbH,Düren, Germany) starting from step six of the manufacturer’s instructions. Elution was performed by adding 100 μL of SE buffer twice. After the DNA extraction, the samples were tested for inhibition by qPCR following the protocol in [33]. Quantitative PCR was performed in duplicate for each sample. If at least one of the replicates showed a different Ct (Cycle threshold) than expected (at least 2 Cts), the sample was considered inhibited and diluted 5-fold before the amplification. The DNA amplifications were performed using “teleo” universal primers following the protocol of [34]. Twelve PCR replicates were performed per field sample. The forward and reverse primer tags were identical within each PCR replicate. This step was conducted in a dedicated room for DNA amplification that is kept under negative air pressure and is physically separated. High-throughput sequencing was carried out on a NextSeq sequencer at Fasteris facilities. Bioinformatic analysis and taxonomic assignment of sequences (for the final taxa list see S1 Table, for details of determination see S1 File) were performed according to [23]. Briefly, the sequence reads were analysed with the OBITools package [35], first the forward and reverse reads were assembled with the illuminapairedend programme. The ngsfilter programme was then used to assign the sequences to each sample. A separate dataset was created for each sample by splitting the original dataset into several files with obisplit. Sequences shorter than 20 bp or occurring less than 10 times per sample or identified as “internal” by the obiclean program were discharged. The ecotag programme was then used for taxonomic assignment with a combination of public available sequences and local database [23]. Given the incorrect assignment of a few sequences to the sample due to tag-jumps [36], all sequences with a frequency of occurrence < 0.001 per sequence and per library were discarded. The data thus obtained were curated for Index-Hopping [37] with a threshold empirically determined for each sequencing batch using experimental blanks (i.e., combinations of tags not present in the libraries) for a given sequencing batch between libraries. Six negative extraction controls and one negative PCR controls (ultrapure water) were amplified with 12 replicates and sequenced in parallel with the samples. It should be noted that a very low number of taxa (2) was detected in the sample from the main Danube channel of the AUT floodplain. This could be related to a major flood in late July that greatly diluted the eDNA in the system [38] or an unnoticed mistake while sampling or during the analysis. Because the number of taxa was significantly lower than the expected one, this sample was discarded from further analysis.

### Data analyses

#### Characterization of spatial and environmental gradients

All statistical analyses were performed in the R environment [39]. First, we categorized the local and regional variables into two explanatory variable groups to examine the relative role of their individual and shared effects on alpha and beta diversity using variance partitioning procedures. These two variable groups were as follows: 1) local variable group including physical and chemical characteristics of the water bodies, which select species based on their niche preferences, exclusively, as well as land use variables, which indicate the effect of the terrestrial realm on aquatic communities 2) regional variable group including hydrological connectivity, which in itself is considered as one of the most important structuring component in the organization of aquatic communities in floodplain ecosystems, and which is also related to spatial positioning of the sampling sites, as well as purely spatial variables (i.e., spatial relationships among the sampling sites) which may indicate the effect of regional scale dispersal limitation among the sampling sites.

We used distance-based Moran’s Eigenvector Maps (dbMEM) to characterize the spatial relationships among the sampling sites. We used the edge-to-edge network distance matrix of the sampled waterbodies as basis, and the function “dbmem” in the package adespatial 0.3.21 with the standard setting for truncation threshold (i.e., the length of the longest edge of the minimum spanning tree) to calculate the spatial variables [40, 41]. We extracted all eigenvalues for further analyses (forward selection and variance partitioning, see below).

We used principal component analyses (PCA) conducted separately on the correlation matrix of the recorded physical and chemical habitat data to characterize both the physical habitat structure and chemical characteristics of the water bodies, using the function “prcomp” in the package factoextra 1.0.7. The advantage of running a PCA on the original environmental data prior to further analysis is that it reduces the number of variables to a small number of largely independent (orthogonal) explanatory variables (here environmental gradients, see e.g. [42–44]). The component scores of the waterbodies along the most influential first two principal components in both the physical habitat structure and chemical PCAs were used as explanatory variables in further analyses (forward selection and variance partitioning, see below). Spearman correlation test was conducted to compute the correlation values between the original environmental variables and the component scores of the sampling sites along the first two PC axes to identify the most influential environmental variables on environmental gradients (PC axes).

As a separate analysis, we used Spearman correlation test to explore the relationship between hydrological connectivity and local scale physical and chemical characteristics of the waterbodies (i.e., PCA axes).

#### Examination of alpha and beta diversity and their determinants

We used general linear models to examine changes in the total number of species (i.e., alpha diversity) and in the number of habitat specialist and generalist species to the hydrological connectivity gradient. Slope of the lines was compared by testing the interaction term of connectivity and species number group types (i.e., total number of species and the number of habitat specialist and generalist species). When the interaction was significant, a Tukey HSD *post hoc* test was used to compare means among the different species number group types.

For beta diversity, total beta was firstly partitioned into species replacement and richness difference components [6] using the function “beta.multi” from the package BAT 2.9.2 [45]. Presence-absence data of the fish community and Jaccard distance were used in the calculation. Distance matrices accounting for the species replacement and richness difference components, as well as their sum (i.e., total beta diversity) were calculated using the function “beta” in the BAT 2.9.2 package. Then we separately applied principal coordinates analysis (PCoA) with Lingoes correction on the three derived matrices (i.e., total beta diversity, richness difference and replacement) [46, 47], using the function “pcoa” in the package ape 5.6.2 [48].

We applied variance partitioning procedures [41] to quantify the pure and shared effects of the two predictor variable groups (i.e., local and regional variable groups) on fish diversity. Before variance partitioning, separate forward selection of the local and the regional variables was conducted using a permutation-based test (function “ordistep” in the package vegan 2.5.7, 999 runs). Only variables significantly (alpha = 0.05) related to community variability were retained in the final models. Partial multiple linear regression and redundancy analysis (RDA) were used for partitioning the explained variance in alpha (i.e., number of species) [49, 50] and beta diversity [41, 51], respectively. For alpha diversity, the analyses were conducted at two levels, at the level of total number of species and at the level of habitat generalist and specialist species. Those taxa which included two or three species belonging to both habitat generalists and specialists were excluded from latter analyses (S1 Table). For beta diversity, the PCoA eigenvectors for species replacement, richness difference and total beta diversity were used as input response variables in separate variance partitioning analyses.

Total variation in diversity was subsequently partitioned into unique and shared contributions of the two sets of predictors, i.e., local and regional variable groups, using adjusted R^2^ statistics [41]. Statistical significance of the unique contributions of the two sets of predictors were tested using the “anova” function in vegan 2.5.7 (999 runs). All analyses were conducted separately for the two sampling occasions.

## Results

### Environmental characteristics of the waterbodies

Hydrological connectivity varied from completely connected (i.e., 100% connectivity with the Danube main channel) to completely isolated sites (i.e., 0% connectivity) (S2 Table).

The physical and chemical PCA analyses characterized well interpretable environmental gradients, at least for their first two axes, which we retained for further analyses (S3 Table). In brief, PC1 of the physical habitat PCA characterized a gradient where relatively deep waterbodies with high velocity, and relatively coarse substrate composition occupied one end, while relatively shallow waterbodies with dense canopy and/or macrophyte cover, woody debris, and fine substrate composition (silt) occupied the other end of the gradient. PC2 of the physical habitat PCA further delineated sites with relatively high canopy cover, and trees along the bank on one end, while relatively dense macrophyte cover and herbaceous bank vegetation at the other end of the gradient (for details see S3 Table). PC1 of the chemical variables PCA showed some differences between different water levels (for details see S3 Table). At high water level dissolved oxygen content and pH correlated negatively, while conductivity correlated positively with PC1. PC2 was characterized by a gradient from low to high primary production and organic particles in the waterbodies, and correlated with chlorophyll-a, CDOM, pH and total P. At low water level dissolved oxygen content correlated negatively while total P and CDOM correlated positively with PC1. Conductivity showed negative while pH, dry weight, ash weight and chlorophyll-a showed positive correlation with PC2.

PC1 and PC2 of the physical habitat PCA showed moderately strong correlations with the hydrological connectivity gradient in both hydrological conditions, while the relationships between connectivity and the chemical PC axes were insignificant (see S4 Table).

### Drivers of alpha diversity

**Fig 3:**
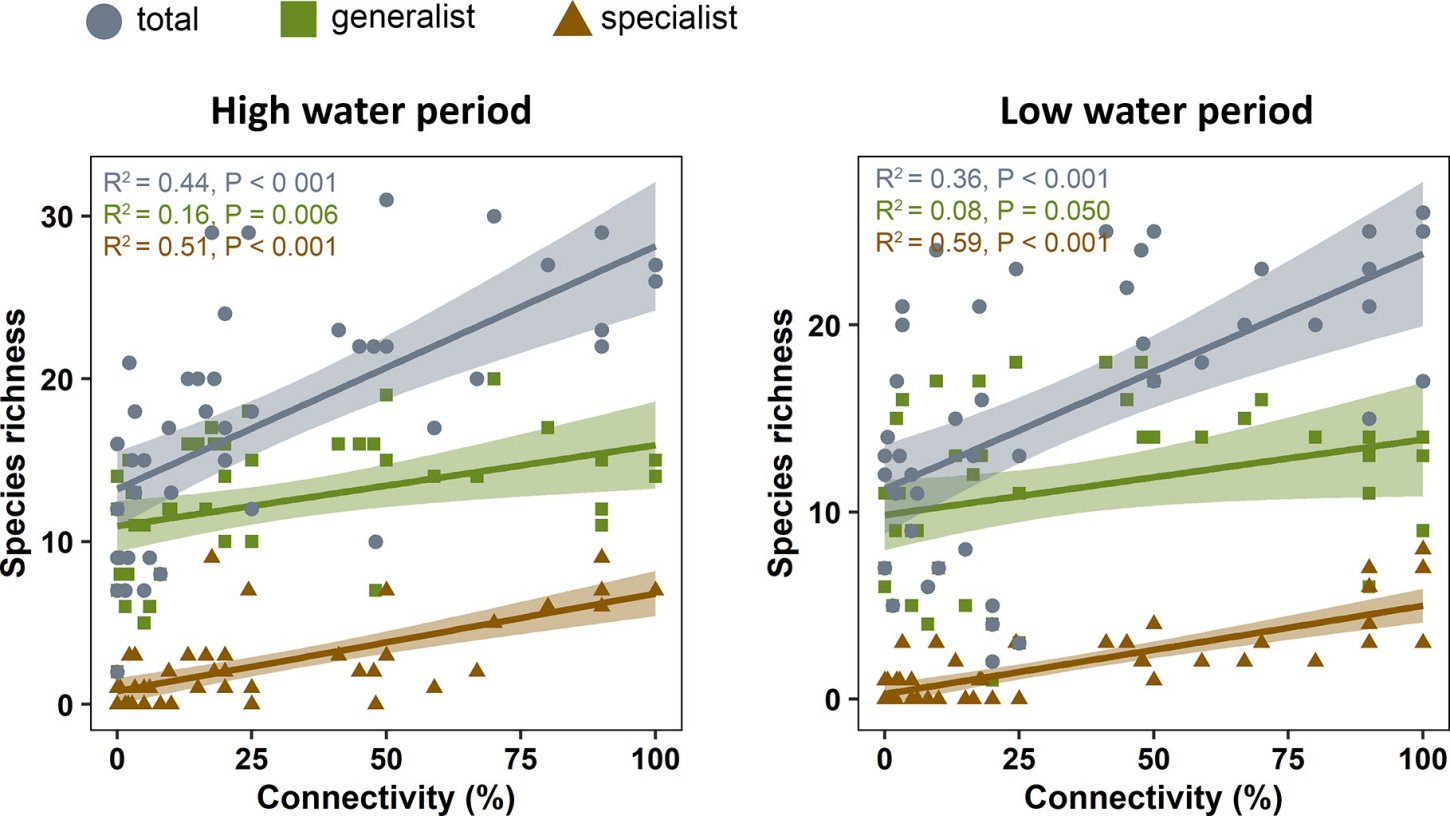
Relationship between species (taxa) richness and hydrological connectivity in high and low water periods. R^2^, P and slope values, and confidence intervals (0.95) around the lines are also shown. Slope of the lines and R^2^ values were calculated using general linear models.

The total number of taxa (hereafter species richness for simplicity) identified with eDNA metabarcoding varied between 2 and 31. Overall, 26 and 13 taxa could be identified as habitat generalist or riverine specialist, respectively (S1 Table). Total species richness and the number of specialist and generalist species increased significantly with increasing connectivity (Fig 3). However, compared to specialists, the relationship of the number of generalist species with hydrological connectivity was much weaker (i.e., R^2^ value was considerably lower for generalist species).

The variance partitioning analyses (Table 1) showed that species richness (alpha diversity) was more effectively predicted by local and regional variables than species composition (beta diversity). The total explained variance for total species richness was 45.8% and 56.9% for the high and low water periods, respectively. Richness of specialist species could be much more effectively predicted (63.1% and 82.0% for the high and low water periods, respectively) than richness of generalists (25.3% and 30.8% for the high and low water periods, respectively). Both purely local and purely regional variables contributed to the explained variance in richness components (Table 1). However, in general, the shared component explained a higher proportion of the variance in richness than the pure fractions (with the exception of generalist richness in the low water period). Additionally, the importance of local scale (environmental) variables was higher in the low water level hydrological period than in the high water period.

**Table 1:** The relative contribution (adjusted R^2^ value %) of purely local (physical and chemical characteristics of the water bodies and land use) and regional (spatial variables and hydrological connectivity) variables, and their shared components on different facets of alpha diversity: Total species number, number of generalist and specialist species; and beta diversity: Total beta and its richness difference and species replacement components at high and low waters. Partial multiple linear regression and redundancy analysis were used for partitioning the explained variance in alpha and beta diversity, respectively. ANOVA was used for testing the statistical significance of the unique contributions of the two sets of predictors. Note that the test cannot be used for the shared components.

		alpha diversity			beta diversity		
		species richness	generalist species richness	specialist species richness	total beta	richness difference	species replacement
high water period	local	6.4*	5.0	11.4**	5.5**	3.9	1.6
	regional	10.4*	7.6*	5.8*	4.5**	5.8*	-
	shared	29.0	12.7	45.9	16.3	17.1	6.2
low water period	local	22.6***	24.5***	13.1***	12.7***	12.3**	5.4**
	regional	5.8*	1.9	17.3***	1.6*	3.1**	0.7
	shared	28.5	4.4	51.6	12.4	14.1	5.3

* P < 0.05

** P < 0.01

*** P < 0.001

### Drivers of beta diversity and its components

Overall beta diversity was moderate and was 0.591 and 0.642 for the high and low water period, respectively (Table 2). The species replacement component of beta diversity was lower than the richness difference component in both hydrological periods.

**Table 2:** Mean values and variance of total beta diversity and its richness difference and species replacement components at high and low water periods in the studied floodplains.

		total beta	richness difference	species replacement
high water period	average	0.591	0.359	0.232
	variance	0.339	0.206	0.133
low water period	average	0.642	0.377	0.265
	variance	0.351	0.206	0.145

The total explained variance for beta diversity was nearly identical in the two hydrological periods; it was 26.3% and 26.7% for the high and low water periods, respectively (Table 1). The richness difference component of beta diversity could be more effectively predicted by the predictor variable groups than the replacement component in both hydrological periods. Like alpha diversity, the shared component was the major determinant of both total beta and its components. Likewise, the importance of local variables was higher in the low water level hydrological period than in the high water period.

## Discussion

We found that the number of generalist species highly outnumbered the number of specialists. We also found that the total number of species decreased from the mainstem river to hydrologically more disconnected sites. Although specialist species responded more strongly to connectivity changes than generalists, we also found that even habitat generalists showed some weak responses to the connectivity gradient. Consequently, both specialist and generalist species influenced the strength of the relationship between connectivity and the total number of species. The direct examination of the relationships among environmental gradients (i.e., PCA axes) and hydrological connectivity showed that the effects of connectivity and physical habitat structure of the waterbodies cannot be separated. Hydrological connectivity influenced physical habitat conditions, thus, not surprisingly, the joint or shared effect of local (environmental) and regional (connectivity and spatial) variables was the major determinant of alpha diversity.

A logical tenet in ecology is that specialist species respond more to specific characteristics of the habitat and are therefore more controlled by niche or species sorting mechanisms than generalist species [8, 28, 52, 53]. Generalist species, on the contrary, respond more to habitat configuration and connectivity, and are therefore more controlled by dispersal or mass effect mechanisms. Our results support this concept only partly since we found the varied effects of both local and regional scale variables on the richness of both specialists and generalists in the two hydrological periods. Rather our study supports other river ecosystem studies, which showed that regional scale hydrological processes shape and interact with local habitat features in river systems, and that the intensity of these relationships varies both in space and time [13, 54]. While other studies also emphasize the role of context dependency in interpreting patterns and processes of river ecosystems [55] including the organization of fish metacommunities [24, 56], this study is the first, to our knowledge, which directly quantifies the variable effects of the different pure and shared variable fractions on the richness of specialist and generalist fishes, and the joint influence of these species groups in shaping both alpha and beta diversity.

Results on beta diversity confirmed our specific hypotheses both on the overall value of beta, and on the relative proportion of its richness difference and replacement components. First, since generalist species outnumbered habitat specialists, the overall value of beta was moderate, despite the fact that we examined diversity patterns along long environmental gradients (i.e., water bodies were represented from the full hydrological connectivity gradient and showed large differences in their physical and chemical characteristics, and land use). Second, richness difference showed consistently higher values than replacement, which is consistent with the hypothesis that the number of riverine specialist species decreases along the connectivity gradient (i.e., isolation from the Danube main channel). In fact, the number of truly lake specialist fishes is very low in Europe due to biogeographic reasons [57, 58], which also contributed to the relatively low value of species replacement along connectivity and habitat gradients in these floodplains.

Although we found a consistent pattern in the relative dominance of richness difference and replacement components between the two examined hydrological periods, other studies suggest high variability in their relative contribution to beta diversity, not only when compared between different realms (e.g., terrestrial and aquatic, see e.g. [17, 53, 59, 60]), but within floodplain metacommunities [12, 13, 61]. For example, studying seasonal changes in a floodplain fish metacommunity Fernandes et al. [13] found that the relative importance of nestedness and turnover can change seasonally. They also found that the relative role of pure and shared components of environmental, spatial and connectivity variable groups varied differentially across the four studied months in explaining the variation in species abundance patterns. Our findings support the theory which posits that at low water periods the importance of local scale habitat variables increases in community structuring relative to high water periods when the importance of mass effect mechanisms increases due to the higher importance of dispersal [62]. Nevertheless, we found only small differences in the values of beta diversity and its components between the two examined hydrological periods, which suggest that the examined yearly flood event influenced fish metacommunity structuring to a lesser extent in this system.

A limitation of the study is that eDNA metabarcoding does not allow the exact identification of some species, which limits the exact determination of the number of habitat specialist vs. generalist species. For example, species of the genus *Gymnocephalus*, which contains both habitat generalist (ruffe, *G*. *cernua*) and specialist species (the yellow pope, *G*. *schraetser* and the Danube ruffe *G*. *baloni*) could not be reliably distinguished based on eDNA. Nevertheless, the number of such unidentifiable species was very low, which surely did not influence the general findings of the study. In fact, even with this limitation, eDNA metabarcoding has been shown to provide a more representative picture on the occurrence of species in this [22, 23] and other systems [20, 63, 64] than traditional fishing methods, and it is especially more suitable for comparing the species composition of waterbodies which differ largely in their environmental features (e.g., flowing vs. standing, deep vs. shallow).

A related issue to the identification of fish taxa using eDNA is that the lateral transport of eDNA fragments across the floodplain could potentially influence patterns of compositional changes when the water bodies are connected to the main channel. However, the hydrological functioning of the studied floodplains and our sampling strategy reduced the risk of false positive detection (i.e., when the species is detected by molecular methods, but in fact it did not occupy the waterbody) to only a few of our studied sites. For sites which are connected to the Danube only during high or very high flow periods we sampled at least two weeks after the disconnection between the main channel and the considered sites, considering that eDNA degrades in less than two weeks for a temperature close to 20°C [65–67]. Nevertheless, most of our study sites were not permanently connected to the main channel even during high water level conditions. Other channels were only connected at its downstream ends to the main channel at low to medium flow, which greatly reduces the possibility for eDNA produced in the main channel to induce false positive detections. Conversely, the eDNA present in a secondary channel permanently connected to the main channel by its two ends can originate partly from the main channel. In this case the risk of false positive detection is mainly a function of the hydrological characteristics of the secondary channel [68, 69], but only a few of our sites belonged to the latter category. In addition, results of our eDNA samplings corresponded well with our former study, which used traditional sampling gears beside eDNA to characterize fish assemblage compositions in these floodplains [22]. Consequently, while some effect of false positive observations cannot be fully ruled out due to the lack of direct observation and identification of species, it is unlikely that such transport mechanisms would significantly determine the observed patterns.

In sum, the results correspond well with our predicted changes in species richness and compositional differences in river-floodplain fish metacommunities along environmental gradients. We found that differences in the number of generalist and specialist species and their responses to connectivity and environmental gradients influenced patterns in alpha and beta diversity. The variance in both alpha and beta diversity could be relatively well predicted by a set of environmental and spatial variables, despite high environmental variability, which characterizes river-floodplain ecosystems. Of these, the joint or shared variance fractions proved to be the most important, which indicates that the effects of local and regional processes cannot be unambiguously separated in these river-floodplain systems. Finally, we found that local scale environmental variables were more important determinants of both alpha and beta diversity in the low water period than in the high water period. These results indicate the differential role of local and regional processes in community organization during different hydrological conditions. Maintenance of both local and regional scale processes are thus important in the preservation of alpha and beta diversity of floodplain fish metacommunities, which should be considered by environmental management.

## Supporting information

S1 Table Fish taxa identified by eDNA metabarcoding and their categorization into habitat generalist or specialist groups based on floodplain fish index habitat affinity scores. (XLSX)

S2 Table Environmental characteristics of the studied waterbodies. (XLSX)

S3 Table Results of PCA conducted on physical and chemical environmental variables. Values indicate Spearman’s rank correlation coefficient between the original environmental variables and the component scores of the sampling sites along the first two PC axes (bold values indicate significant (P<0.05) correlation).
(XLSX)

S4 Table Spearman’s rank correlation coefficient between hydrological connectivity and the component scores of the sampling sites along the first two PC axes. PCAs were conducted separately on the physical and chemical environmental variables.
(XLSX)

S1 File Details of the determination of taxonomic status based on the output of eDNA sequencing and bioinformatic analysis. (DOCX)
