# Supplementary material for: eDNA metabarcoding reveals the role of habitat specialization and spatial and environmental variability in shaping diversity patterns of fish metacommunities
Source: PLoS One. 2024 Jan 2;19(1):e0296310. doi: 10.1371/journal.pone.0296310 (PMC10760726; doi:10.1371/journal.pone.0296310)
Supplement: S1 File — (DOCX) [file pone.0296310.s005.docx]

**Details of the determination of taxonomic status based on the output of eDNA sequencing and bioinformatic analysis.**

Reference sequences assigned at the family level were discarded (Acipenseridae, Cyprinidae).

References sequences assigned at the genus level were retained when there was no assignment at a lower taxonomical level (*Coregonus spp., Sander spp., Sardinella spp., Scomber spp., Trachurus spp.*)

When reference sequences were assigned at the genus level, they were ultimately named to the species level when only one species from the genus was known in the catchment (*Anguilla Anguilla, Perca fluviatilis*).

When, for a given genus with multiple species known to occur, reference sequences were assigned at both genus and species level, the genus-level references were discarded (*Acipenser spp, Alburnus spp., Ameiurus spp., Barbus spp., Carassius spp., Leuciscus spp., Romanogobio spp.*)

Molecular markers failed to discriminate species for some groups with two to three species present in the catchment. These groups were as follows:

*Abramis sapa, Blicca bjoerkna* and *Vimba vimba* (Cyp_1)

*Chondrostoma nasus* and *Telestes souffia* (Cho_nas)

*Gobio gobio, Romanogobio kessleri* and *Romanogobio vladykovi* (Cyp_2)

*Gymnocephalus baloni, Gymnocephalus cernua* and *Gymnocephalus schraetser* (Cyp_3)

*Ctenopharyngodon idella, Hypophthalmichthys nobilis* and *Hypophthalmichthys molitrix*

*Leuciscus idus, Leuciscus leuciscus* and *Pelecus cultratus* (Cyp_4)

*Rutilus virgo* and *Rutilus pigus* (Rut_vip)

*Salvelinus fontinalis* and *Salvelinus alpinus* (Sal_1)

*Sander lucioperca* and *Sander volgensis*

Because *Telestes souffia* is a species well known to occur primarily in upstream fast-flowing river reaches and has not been previously caught [1] or eDNA-detected in the Danube River [2], we considered the occurrence of the corresponding group to *Chondrostoma nasus*.

When species belonging to one these groups were also assigned at the species level (*Blicca bjoerkna, Leuciscus idus, Romanogobio kessleri*), their corresponding number of reads was cumulated.

After the final taxonomic identification of sequences, several categories of taxa were considered.

The first category included all taxa confirmed to occur in the Danube River (Known-taxa) by previous traditional fish sampling surveys [1, 3] or by the literature [4-6].

The second category (Waste-taxa) included food fish, farmed fish, aquarium fish or fish with any other link to human activity allowing a rejection of extra-organism eDNA in the river (mainly sewage): Ammodytidae, *Clarias gariepinus*, *Clupea harengus, Eleotris acanthopoma, Istiophorus albicans, Merlangius merlangus, Micromesistius poutassou, Sardina pilchardus, Sardinella longiceps, Sardinella sp., Scomber sp., Trachurus sp*.

The third category included species (*Leuciscus burdigalensis, Oncorhynchus gorbuscha*) unknown in the catchment and not known for any human use (Unknown-taxa).

*Oncorhynchus mykiss, Salmo trutta, Salvelinus namaycush* and *Salvelinus spp*. occur in fast flowing sections of the Danube tributaries and alpine lakes but are also known as very common food fish. As they are never or very rarely caught in the Danube River itself [1, 3], the detection of their eDNA in our sampling sites should be interpreted with caution and we included them to the Waste-taxa category.

Based on these considerations, the final list of taxa that were used for the analyses can be found in Table S1.

**References**

1 Erős, T. *et al.* Typology of a great river using fish assemblages: implications for the bioassessment of the Danube river. *River Res. Appl.* **33**, 37-49 (2017).

2 Pont, D. *et al*. Quantitative monitoring of diverse fish communities on a large scale combining eDNA metabarcoding and qPCR. *Mol. Ecol. Resour*. **23**, 396– 409 (2022).

3 Bammer, V. *et al.* (Eds.), *Joint Danube survey 4 scientific report: A shared analysis of the Danube River* (pp. 41–54). ICPDR Ed.

4 Kottelat, M. & Freyhof, J.. *Handbook of European Freshwater Fishes*. Publications Kottelat, Cornol, Switzerland and Berlin, Germany (2007).

5 Meulenbroek, P. *et al.* Species-specific fish larvae drift in anthropogenically constructed riparian zones on the Vienna impoundment of the River Danube, Austria Species occurrence, frequencies, and seasonal patterns based on DNA barcoding. *River Res. Appl.* **34**, 854–862 (2018).

6 Sommerwerk, N. *et al.* *The Danube River basin*. In K. Tockner, C. Zarfl, & C. Robinson (Eds.), Rivers of Europe (pp. 59–112). Elsevier Academic Press (2009).
